# Trace Element Composition of Surface Water in Almaty City and Human Health Risk Assessment

**DOI:** 10.3390/ijerph21111511

**Published:** 2024-11-14

**Authors:** Marina Krasnopyorova, Igor Gorlachev, Pavel Kharkin, Dmitriy Zheltov, Mariya Severinenko, Adilzhan Serikov

**Affiliations:** Institute of Nuclear Physics, Ibragimov 1, Almaty 050032, Kazakhstan

**Keywords:** water, urban rivers, heavy metals, water quality indices, carcinogenic risk, non-carcinogenic risk

## Abstract

This investigation meticulously examined the elemental composition of 64 water samples collected during the seasons of spring, summer, autumn, and winter of the year 2023. The average seasonal concentrations of arsenic (As), beryllium (Be), cobalt (Co), cadmium (Cd), copper (Cu), lithium (Li), molybdenum (Mo), nickel (Ni), lead (Pb), selenium (Se), uranium (U), mercury (Hg), aluminum (Al), barium (Ba), chromium (Cr), iron (Fe), manganese (Mn), strontium (Sr), vanadium (V), zinc (Zn), calcium (Ca), potassium (K), magnesium (Mg), sodium (Na), and chlorine (Cl) as well as SO_4_ and dry residue were computed at 16 strategically selected sites along the Bolshaya and Malaya Almatinka, Esentai, and Kargalinka rivers situated in Almaty. The sampling locations were categorized into three distinct sectors: upper (adjacent to mountainous regions), middle (urban zone), and lower (exceeding city limits), thereby facilitating the examination of discrepancies in water quality and elemental concentrations. The results reveal that surface water resources in Almaty, particularly concerning As, Ni, Cr, U, and Pb, may present a considerable carcinogenic risk if utilized for consumption purposes. This is especially alarming given that these rivers constitute a vital source of drinking water for the inhabitants of the city. Specifically, at two sampling locations along the Bolshaya and Malaya Almatinka rivers in proximity to significant urban thoroughfares, untreated river water displayed an elevated carcinogenic risk (CR ~ 10−^2^). These results highlight the urgent necessity for enhanced water treatment and ongoing monitoring to safeguard public health.

## 1. Introduction

The pollution of aquatic ecosystems with heavy metals (HMs) presents a significant concern due to their inherent toxicity and propensity for accumulation within aquatic environments [1,2]. In contemporary society, there has been a pronounced and concerning escalation in HM pollution levels, which has emerged as a pressing environmental challenge necessitating immediate intervention and remedial measures [3,4,5]. For instance, the comprehensive review conducted by Vinod Kumar et al. evaluates the concentration of HMs in global surface water bodies from 1994 to 2019. Within the scope of the 147 publications analyzed, the average concentrations of Cr, Mn, Co, Ni, As, and Cd surpassed the permissible thresholds established by the WHO and USEPA. Findings derived from the heavy metal pollution index, evaluation index, pollution degree, water contamination, and toxic load indices suggest that the examined water bodies are extensively contaminated with HMs [6]. Li et al., in their investigations regarding HM pollution in China, indicated that several urban areas with high population densities, including Kunming, Zhuzhou, Chenzhou, Shaoguan, and Chongqing, are significantly polluted and necessitate targeted HM management strategies to mitigate their effects on human health [7]. They further observed that elevated HM levels in aquatic environments are predominantly linked to anthropogenic activities such as urban expansion and industrial practices. Qu et al. documented concentrations of As (1.71 µg/L), Cr (7.7 µg/L), Pb (30.1 µg/L), Cd (16.0 µg/L), Hg (46.1 µg/L), Cu (96.8 µg/L), and Zn (98.3 µg/L) in the Wen-Rui Tang River in China, attributing the high HM levels primarily to the electroplating and tanning sectors [8]. Kumar et al., in their examinations of Indian river systems, demonstrated that the upper stretches exhibit lower pollution levels in comparison to the lower reaches [9,10]. This phenomenon may be a result of industrial and sewage effluents in the upper areas accumulating towards the lower sections. Pekey et al., in their analysis of the DilDeresi stream, identified that wastewater from Turkey’s paint and coating industries is the principal contributor to heavy metal pollution in the stream. It is estimated that approximately 60% of the total wastewater and 80% of the total organic carbon flow directly into Izmit Bay via the DilDeresi stream [11]. Edris Bazrafshan et al. investigated the concentrations of eight heavy metals (Cr, Cd, Cu, Mn, Fe, Pb, Zn, and Ni) in the surface waters and sediments of the Chah Nimeh reservoir. Their results indicated that the overall concentrations of heavy metals in both water and sediments did not surpass WHO guidelines (with the exception of Cd). The presence of heavy metals in surface waters and sediments is attributed to the discharge of municipal effluents and the runoff of agrochemicals and fertilizers from adjacent villages, either directly or indirectly, into the reservoir [12].

The body of scientific literature encompasses a plethora of studies addressing heavy metal contamination in water resources [13,14,15,16]. Collectively, these researchers affirm that consistent monitoring of water quality is imperative, as the rising levels of heavy metals in river water pose an escalating threat to both human health and agricultural productivity.

The human organism necessitates specific trace metals (such as copper, zinc, and iron) for intracellular functions and DNA-binding activities. The majority of these metals exhibit toxicity, even at minimal concentrations, and possess carcinogenic properties. Under specific environmental circumstances, heavy metals can accumulate to hazardous concentration levels, leading to considerable environmental repercussions and detrimental effects on human health [17,18]. The negative implications of heavy metals are extensive, as they can adversely affect nearly all physiological systems within the human body, resulting in a range of harmful effects, including toxicity, allergic responses, potential carcinogenicity, and alterations in gonadotropic functions [19].

Almaty represents the largest urban center in Kazakhstan. The predominant environmental challenges faced by the city are associated with anthropogenic factors characteristic of extensive megacities (thermal power facilities, industrial enterprises, irregular high-rise urban development, vehicular traffic, etc.). The principal contaminants in the natural environment of the city comprise phenol, carbon monoxide, nitrogen dioxide, particulate matter, and heavy metals. Almaty is situated within a natural depression where phenomena such as fog, lack of wind, and surface inversions are prevalent, thereby complicating the spatial dispersion of pollutants. Under generally favorable climatic conditions, the foothill zone of the Zailiyskiy Alatau exhibits limited atmospheric self-purification capabilities. The urbanization and industrial growth of the city transpired without adequate regard for the natural–ecological equilibrium and physiographic attributes of the megacity. Furthermore, as indicated by previous investigations, elevated concentrations of uranium have been detected in the drinking water samples of the city. This is presumably attributable to the leaching of chemical elements from the geological formations, where they exist in a diffuse state. The elemental composition of surface watercourses sampled seasonally in Almaty was examined as part of the research endeavor. Utilizing the acquired data, the seasonal mean concentrations and standard deviations for all sampling sites were calculated. The concentrations of chemical elements found in the water samples were compared with the maximum permissible levels for potable water, and the water use classification for each sampling location was established. Based on the findings obtained, the carcinogenic and non-carcinogenic risks associated with individual heavy metals entering the human body via dermal contact, accidental ingestion of water, and the utilization of water from the analyzed rivers for drinking purposes were evaluated.

## 2. Study Area

The urban locality of Almaty is strategically situated at the center of the Eurasian landmass, specifically in the southeastern region of the Republic of Kazakhstan. Its precise geographical coordinates are delineated as 77 degrees east longitude and 43 degrees north latitude. Almaty is aesthetically positioned in the foothills of the Zailiysky Alatau, recognized as the northernmost mountain range within the Tian Shan system. The aggregate area encompassed by the city exceeds 170 square kilometers. It is positioned within the valley formed by the Bolshaya and Malaya Almatinka rivers and their tributaries, which originate from the glacial formations of the Zailiysky Alatau and its mountainous gorges. The primary sources of potable and domestic water for Almaty are derived from mountain rivers, lakes, and groundwater.

The Bolshaya Almatinka River extends for 29 km. This river originates from the slopes of the glaciers of the Zailiysky Alatau at an elevation of 3500 m. The total length of this river measures 96 km, with its catchment area encompassing 425 square kilometers. The river is constituted by the confluence of three streams that arise from the moraines of two glaciers located in the Zailiysky Alatau. It is susceptible to occurrences of mudflow.

The Malaya Almatinka River, measuring 28 km in length, undergoes variations in water levels, primarily influenced by atmospheric precipitation and groundwater contributions. Its source can be traced to the Tuyuksu glaciers situated in the Zailiysky Alatau region. The cumulative length of this river is 125 km, with a corresponding catchment area of 710 square kilometers. This river exhibits a particular vulnerability to mudflow incidents.

The Yesentai (Vesnovka) River, extending 25.1 km, serves as a left tributary of the Malaya Almatinka River. Its overall length totals 43 km, and it is categorized as being at risk of mudflows.

The Kargalinka River spans 57 km in length and possesses a catchment area of 98 square kilometers. This river is nourished by 15 minor tributaries that emanate from springs, collectively extending 27 km. The width of the riverbed varies between 5 and 10 m, with a depth ranging from 0.3 to 0.5 m, occasionally reaching up to 1 m in specific locations. The Kargalinka basin is situated within the middle and lower mountainous zones of the western segment of the Zailiysky Alatau.

As water traverses the slopes from the watershed regions, it becomes increasingly enriched with various ions derived from highly soluble minerals present in the geological formations. The extent of mineralization of the water is positively correlated with the distance traveled through these rocks. The intrinsic structure of the rocks dictates the predominant size of the resultant weathering products. Massive geologic formations (such as igneous rocks, sandstones, and certain limestones) weather into substantial fragments, while thinly layered marls and shales disintegrate into pebble-boulder aggregates. The chemical composition of mountain rivers is also influenced by the processes of leaching or chemical weathering of the surrounding rocks.

As waterways traverse the urban landscape, an exacerbation of pollution is instigated by anthropogenic waste. The principal contributors to water contamination in Almaty comprise the pulp and paper manufacturing sector, light industrial activities, municipal services, thermal power generation facilities (TPPs), and diminutive boiler installations. At present, the Almaty agglomeration is regarded as one of the most ecologically compromised regions within Kazakhstan, with all natural habitats severely contaminated by hazardous chemicals.

Consequently, the concentration of trace elements within the rivers of the mid-mountain region is affected by both geological substrates and polluted atmospheric precipitation, in addition to anthropogenic activities occurring within the watershed areas. In light of this understanding, we categorized the sampling locations into three distinct classifications. Sites situated in proximity to the mountain range are associated with natural pollution attributable to the interaction with geological materials. The second classification encompasses sites located in the highly urbanized central sector of the city. This area is characterized by substantial vehicular traffic and a dense concentration of residential complexes, which are likely to exert a significant influence on the elemental composition of the riverine water. The third classification pertains to the lower section of the city, where the river channel exits the urban sphere. In this locale, while human activity diminishes, substantial industrial facilities, storage warehouses, and metal recycling operations are prevalent, which may also affect the elemental composition of the river. This classification framework enables the monitoring of variations in the concentrations of specific chemical elements as well as the overarching water quality of Almaty’s rivers as they navigate through the urban environment.

## 3. Materials and Methods

### 3.1. Sampling and Sample Preparation

**Figure 1:**
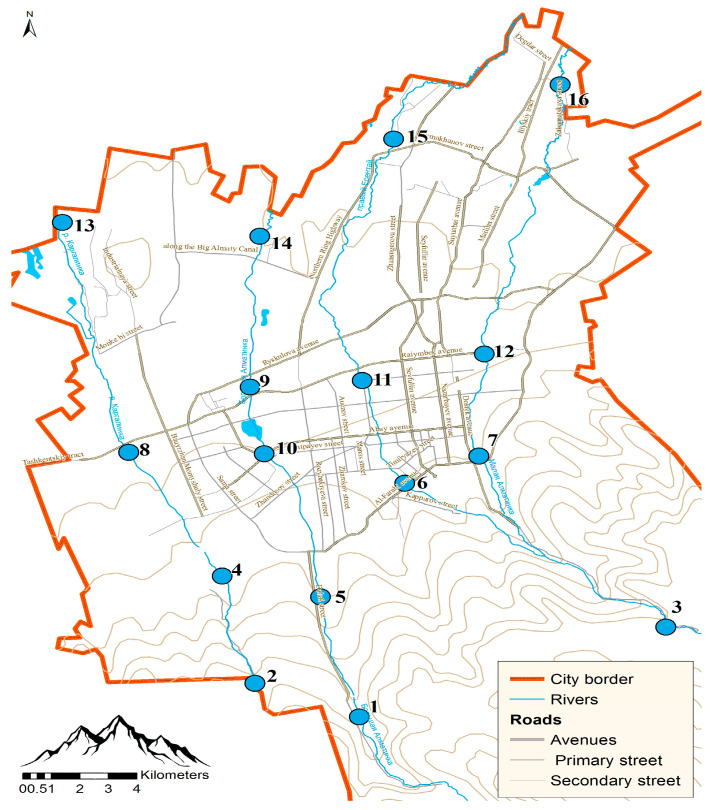
Water sampling points located along the course of rivers in Almaty city.

Figure 1 shows a map of Almaty City with the main surface watercourses marked and 16 water sampling points. To study the elemental composition, 64 water samples were collected in winter, spring, summer, and autumn 2023, with 16 samples each season.

The sampling points have the following geographic coordinates: Point 1: 43°07′45.8″ N, 76°54′28.9″ E; Point 2: 43°08′36.2″ N, 76°51′47.9″ E; Point 3: 43°09′45.1″ N, 77°02′30.0″ E; Point 4: 43°11′08.9″ N, 76°51′01.6″ E; Point 5: 43°10′37.1″ N, 76°53′33.7″ E; Point 6: 43°13′15.7″ N, 76°55′50.3″ E; Point 7: 43°13′52.5″ N, 76°57′46.8″ E; Point 8: 43°14′06.7″ N, 76°48′40.9″ E; Point 9: 43°15′35.9″ N, 76°51′53.2″ E; Point 10: 43°14′01.3″ N, 76°52′12.5″ E; Point 11: 43°15′42.5″ N, 76°54′48.2″ E; Point 12: 43°16′16.8″ N, 76°58′00.2″ E; Point 13: 43°19′33.8″ N, 76°47′06.9″ E; Point 14: 43°19′09.6″ N, 76°52′14.8″ E; Point 15: 43°21′23.6″ N, 76°55′48.3″ E; Point 16: 43°22′36.1″ N, 77°00′10.7″ E.

Water sampling was executed in accordance with the stipulations outlined in normative documentation [6]. The sampling procedure utilized disposable polyethylene containers of laboratory-grade HDPE that were sealed with tightly fitting lids. Prior to the introduction of the water sample into the container, it underwent a rinsing process three times with the same water. Documentation concerning the geographical location and environmental conditions of the sampling was provided through labels affixed to the container.

Within the analytical laboratory, the removal of suspended solids, sediments, and mechanical impurities from the collected water samples was achieved through filtration utilizing a “White Ribbon” paper filter. The analysis of the elemental composition was performed without any dilution of the samples 24 h after the sampling event.

### 3.2. Methods of Analysis

#### 3.2.1. ICP-MS and ICP-AES

A comprehensive approach was used to determine the heavy metals in the natural water, including the following techniques:

Inductively coupled plasma mass spectrometry was first used. For the ICP-MS method, we used the quadrupole mass spectrometer ELAN-9000 (Perkin Elmer Corporation, Concord, ON, Canada), which has a standard deviation of output signal less than 6%, resolution from 0.6 to 0.8 atomic mass units at 10% peak height and a range of analysed masses from 2 to 270 atomic units [7,8].

Optical emission spectrometry with inductively coupled plasma was also used [9]. For the ICP-AES method, a double-view optical emission spectrometer OPTIMA-8000 (Perkin Elmer Corporation, Singapore) capable of operating in the optical range from 166 to 900 nm with a half-peak resolution of 0.008 nm at 200 nm was used.

The analyzed isotope (for ICP-MS) and emission line (for ICP-AES) were selected based on a criterion that compromises the acceptable sensitivity of element content determination, minimum spectral interference, and low background level.

Diluted standard samples with metal compositions of 10 µg/L and 100 µg/L produced by Inorganic Ventures, Christiansburg, Virginia, USA were used for the ICP-AES calibration.

In order to correct for instrumental drift, internal standards at the rate of 5 µg/L Rh (for ICP-MS) and 0.25 mg/L Sc (for ICP-AES) were introduced into all measured samples and calibration solutions. To check the reproducibility of the analysis, measurements of duplicate samples prepared along with the analyzed samples were performed. To verify the accuracy of the analysis and the effect of the sample matrix on the analysis results, measurements of spiked samples (with known additions) were conducted. Analysis of the total content of non-volatile minerals and partially organic compounds dissolved in water was carried out according to the standard [10]. Water samples were heated to boiling point in a special vessel until complete evaporation, after which the sediment at the bottom of the vessel was weighed. The result is expressed as the mass fraction of the dry residue as a percentage of the volume of the original water.

The gravimetric method for determining the sulfate content in natural waters is based on weighing the precipitate obtained after treatment of a water sample with barium chloride [11].

The chloride ion content was determined using the titrimetric method based on the use of mercaptobenzothiazole-trifluoroacetic acid by controlling the color change of the formed complex [12].

#### 3.2.2. Water Quality Assessment

Two approaches were considered to assess the surface water quality in terms of the chemical elements contained therein.

In accordance with the normative document adopted in the RK [13], water bodies were classified into six quality classes depending on the degree of pollution (Table 1, three classes are indicated). Table 2 lists the threshold values of the chemical element content for each class.

The methodology delineated in a normative document [13] has been implemented in Kazakhstan to evaluate the appropriateness of aquatic systems for potable usage. Nevertheless, it functions solely with threshold characteristics, failing to encapsulate the comprehensive extent of water suitability. Alternate methodologies exist for the evaluation of water quality.

The weighted arithmetic water quality index method (WAWQIM), formulated by Brown et al. [14] and endorsed by the World Health Organization, quantifies the assessment of water quality, thereby elucidating the extent of its fitness for drinking applications. In this paradigm, the water quality index (WQI) serves as an aggregate representation of the influence exerted by all parameters on the cumulative water quality [15]. The arithmetic weighted water quality index method is applicable across both surface water and groundwater contexts [16]. Presently, this methodology is extensively utilized by a diverse cadre of researchers [17,18,19,20].

The weighted arithmetic water quality index method is predicated upon the computation of the cumulative ratios of the concentrations to the maximum permissible concentrations (MPC) for each water parameter under examination. In this analytical framework, a weighting coefficient was ascribed to each parameter, calculated in accordance with the maximum permissible concentration associated with that parameter. The lower the MPC, the greater the significance attributed to this parameter in the computation of the WQI.

(1) $$WQI=\frac{\sum_{i=1}^{n}Q_{i}W_{i}}{\sum_{i=1}^{n}W_{i}}$$

(2) $$Q_{i}=\frac{C_{i}}{S_{i}}100\%$$

where

Ci—concentration of i-th element in water sample,
Si—maximum permissible concentration of the i-th element in drinking water.

The specific weight (Wi) of each water quality parameter was calculated using the following formula:

(3) $$W_{i}=\frac{K}{S_{i}}$$

where K is the proportionality factor, which can be calculated using the following equation:

(4) $$K=\frac{1}{\sum_{i=1}^{n}\left(\frac{1}{S_{i}}\right)}$$

The water quality rating according to the WQI is summarized in Table 3.

#### 3.2.3. Calculation of Carcinogenic and Non-Carcinogenic Risks

The ultimate aim of the environmental quality assessment is to ascertain the influence of pollutants on human health, specifically through risk assessment methodologies. The research presented herein evaluates both non-carcinogenic and carcinogenic risks associated with the typical bathing practices of an average adult in the analyzed rivers (external exposure, dermal), the incidental ingestion of water (internal exposure, ingestion), and the consumption of this river water (internal exposure, oral). The elements considered as primary determinants include As, Co, Cd, Cu, Mo, Ni, Pb, U, Cr, Fe, and Mn. In evaluating the carcinogenic risk associated with external exposure, the concentrations of As, Cd, Ni, Pb, and Cr in the water were analyzed; conversely, for internal exposure via ingestion, the elements As, Cd, Ni, Pb, Cr, along with U due to its radiotoxic characteristics, were accounted for.

The calculation methodologies employed were aligned with the international guidelines established by the United States Environmental Protection Agency (USEPA) [21,22,23,24]. This methodological framework is employed to ascertain the characteristics and magnitude of health risks linked to chemical pollutants. The guidelines established by the USEPA delineate the principal strategies utilized for the identification and quantification of risks, alongside the techniques and reference benchmarks for evaluating threshold hazard levels (dosage).

Daily exposure dose:

(5) $${{CDI}_{i}}_{dermal}=C_{i}\times \frac{SA\times Kp\times ET\times EF\times ED\times CF}{BW\times AT}$$

(6) $${{CDI}_{i}}_{ingestion/oral}=C_{i}\times \frac{IR\times EF\times ED}{BW\times AT}$$

where:

CDIidermal—daily exposure dose of the i-th element upon contact with skin (μg/(kg × day));
CDIiingestion/oral—daily exposure dose of the i-th element when swallowed or drunk ((µg/(kg × day));
C_i_—concentration of the i-th element in water (μg/L);
SA—exposed body surface (sm^2^);
Kp—skin permeability coefficient (sm/h);
ET—contact time (h/day);
CF—unit conversion factor (L/sm^3^).
BW—body weight (kg);
AT—exposure period (day);
IR—volume of water intake (L/day);
EF—frequency of exposure (day/year);
ED—duration of exposure (year).
Non-carcinogenic risk:

(7) $${HQ}_{i}=\frac{{CDI}_{idermal/ingestion/oral}}{Rf{Di}_{dermal/ingestion/oral}}$$

(8) $$HI=\sum {HQ}_{i}$$

where:

HQ_i_—hazard quotient of the i-th element for intake by different paths;
RfD_i_—reference dose values (µg/(kg × day));
HI—total hazard index;
HI < 1 corresponds to minor health impact;
HI ≥ 1 indicates the existing risk [25]. 1 < HI ≤ 5 low risk, 5 < HI ≤ 10 medium risk, HI > 10 high risk [26].

The safe level for a single chemical element is HQ < 1.

Carcinogenic risk:

(9) $${{CR}_{i}}_{dermal/ingestion/oral}={CDI}_{i}\times {SF}_{i}$$

(10) $$CR=\sum {CR}_{i}dermal+\sum {CR}_{i}ingestion+\sum {CR}_{i}oral$$

where:

CR_i_—carcinogenic risk index of the i-th element for intake by different paths;
SF_i_—slope factor (µg/(kg × day)^−1^;
CR is classified as follows:
CR < 1 × 10^−6^—there is no risk;
1 × 10^−6^ < CR < 1 × 10^−4^—acceptable level of risk;
CR > 1 × 10^−4^—risk is unacceptable [27].

The reference dose value (RfD) and slope factor (SF) are measures of uncertainty in assessing non-carcinogenic and carcinogenic risks. The RfD is a conditionally acceptable dose of an adverse health effect, and the greater the excess of the exposure dose over the acceptable dose, the more adverse the effect is expected to be. The SF is the degree of increase in the probability of developing cancer when exposed to a carcinogen. The SF is defined as the upper 95% confidence limit of the slope of the dose–response relationship in the lower linear portion of the curve.

The parameters for the calculation, as well as the sources of the accepted data, are given in Table 4.

The carcinogenic potential associated with exposure to uranium was determined through an evaluation of its radiotoxic characteristics. The primary naturally isotopes of uranium consist of U-238 and U-234.

Uranium-238 constitutes the predominant fraction (99.3%) of the total mass of naturally occurring uranium. Concurrently, its progeny isotope, Uranium-234, is characterized by a higher emission of alpha radiation compared to Uranium-238 [34]. Consequently, in the evaluation of radiotoxic properties, it is prudent to consider the coexistence of these two isotopes within chemically defined uranium. This evaluation is grounded in the perspectives articulated in a multitude of scholarly publications [35,36,37,38]. The computation of surplus radiological risk is executed utilizing the following formula:

(11) $${CR}_{U}=A_{U}\times r\times I$$

where:

A_U_—total activity of isotopes U (U-238 + U-234) (pCi/L);
r—risk coefficient;
I—the amount of water consumed during a human life (L), calculated as IR × EF × ED (Table 4), which was 67.50 L when swallowed and 56.210 L when drunk.

The conversion factor for the mass content of 1 μg/L to activity (A_U_) is 0.67 pCi/μg [39], based on the fact that 1 g of uranium contains 12.356 Bq U-238 and 12.356 Bq U-234 [40]. The risk coefficient r is expressed as the risk of mortality (1.13 × 10^−9^) from cancer or morbidity (1.73 × 10^−9^) per unit of consumed activity (Bq^−1^). In our case, the risk coefficient r is replaced by SF, similar to [31] 6.40 × 10^−11^ (risk/pCi) for U-238 and 7.07 × 10^−11^ (risk/pCi) for U-234, which is recommended by USEPA [33]. SF for the final risk assessment is adopted for the most abundant U-238.Thus,

(12) $${CR}_{U}=C_{U}\left(\frac{µg}{L}\right)\times 0.67\left(\frac{pCi}{µg}\right)\times {SF}_{U}\left(\frac{Risk}{pCi}\right)\times I(L)$$

The acceptable value for the lifetime risk of cancer from uranium exposure is CR_U_ < 10^−6^ [31,32].

## 4. Results and Discussion

### 4.1. Elemental Composition Analysis

The analytical techniques of inductively coupled plasma mass spectrometry (ICP-MS) and inductively coupled plasma atomic emission spectroscopy (ICP-AES) were employed to ascertain the concentrations of 26 distinct chemical elements within water samples procured during the winter, spring, summer, and autumn seasons of the year 2023 across each designated sampling site.

Utilizing the acquired data, the arithmetic means and standard deviations were computed for each sampling location. Table 5 delineates the seasonal averages (mean) and standard deviations (SD) for those elements whose mean concentrations surpassed the detection limit (LD) established by the employed methodologies. Furthermore, the table enumerates the maximum permissible concentrations and associated hazard classifications [41]. In all analyzed samples, the concentrations of the elements arsenic, beryllium, cadmium, selenium, vanadium, and mercury did not surpass the detection limits as defined by the methodology, which are 50 µg/L for As, 0.2 µg/L for Be, 1 µg/L for Cd, 10 µg/L for Se, 100 µg/L for V, and 0.5 µg/L for Hg. Regarding uranium, calcium, magnesium, and potassium, there exists no designation of maximum permissible concentrations (U and K) or hazard classifications (K, Ca, Mg, and U) within the extant regulatory literature.

Upon examination of the data presented in Table 5, it is evident that the exceedance of the maximum permissible concentration (MPC) for barium (Hazard Class 2) is recorded at sampling point 9 (the middle stretch of the Bolshaya–Almatinka River), and for calcium, it is recorded at sampling locations 1, 12, and from 13 to 16. The barium concentration at sampling point 9 (illustrated in Figure 1) during the spring sampling was quantified at 320 µg/L. Notably, such elevated concentrations of this element were not observed at this sampling point during other seasonal assessments. Similarly, the adjacent sampling points during the spring collection (points 10 and 14) also did not exhibit typically high barium concentrations. It may be postulated that there exists localized seasonal contamination within a specific section of the Bolshaya–Almatinka River. Elevated levels of barium at specific sampling locations may be correlated with concentrated industrial effluents or temporal variations in agricultural runoff, especially in relation to the phenomenon of spring thaw.

Calcium concentrations surpassed the MPC during the winter sampling at points 1, 4–6, and 10–15, effectively encompassing nearly all segments of the rivers under investigation. For spring and summer sampling, exceedance was particularly noted at point 16, while for autumn sampling, points 1, 6–7, and 11–16 exhibited exceedance. It is plausible that the calcium concentration in surface water samples is influenced by seasonal variations. Additionally, an exceedance of the MPC for chloride ions was recorded at point 13, and at point 16 for magnesium and sulfate ions. Elements such as calcium and magnesium may exhibit seasonal variations attributable to alterations in hydrological flow patterns and precipitation, which can enhance the leaching processes of minerals from adjacent soils and mountainous regions. Anthropogenic activities, including irrigation practices, agricultural runoff, and heightened industrial operations during warmer seasons, may provide further elucidation for the variations in elemental concentrations across different times of the year.

At sampling locations 14, 15, and 16, which correspond to the lower segments of the river, elevated concentrations of cobalt, lithium, molybdenum, nickel, uranium, strontium, and zinc are documented in comparison to other urban locales. This area accommodates substantial industrial entities, storage facilities, and metal recycling operations, which may significantly affect the elemental profiles of the river systems. Increased concentrations of lead, manganese, barium, and zinc are noted in the central urban area sampling points 5, 6 and 7, which is characterized by the highest traffic density and the greatest number of residential complexes, likely influencing the elemental makeup of the aquatic systems in these regions. Ultimately, the upper section of the city exhibits the most pristine river water concerning elemental composition. Nevertheless, at sampling point 1, heightened uranium concentrations are detected, likely resulting from natural leaching processes from the surrounding mountainous terrain, potentially intensified by climatic conditions or geological characteristics.

The uranium concentrations detected in our investigations are noteworthy, with levels in certain areas surpassing 14 µg/L, which exceeds the conventional global benchmarks for natural waters (typically below 4 µg/L in river systems and 3.3 µg/L in marine environments [42]). In relation to the World Health Organization (WHO) guideline stipulating a maximum uranium concentration of 30 µg/L in potable water, these findings raise alarm, particularly in regions where concentrations approach this critical limit. Prolonged exposure to uranium in drinking water, even at these comparatively lower levels, may pose substantial health risks due to its chemical toxicity and radiological implications, predominantly affecting renal function. The data imply that the presence of uranium in the surface waters of Almaty, especially in the upper sections of the city, may be associated with natural leaching from geological formations; however, it remains a pressing public health issue that necessitates ongoing surveillance and strategic mitigation measures.

Concurrently, such anomalies are not exclusive to this study. For instance, an analysis of 476 groundwater samples from Norway revealed that in 18% of these instances, uranium concentrations exceeded 20 µg/L [43]. Concentrations surpassing 20 µg/L have also been documented in groundwater from various regions within New Mexico, USA [44,45].

### 4.2. Water Quality Index Calculation

The classification of water quality was ascertained for each sampling location in accordance with the criteria delineated in Table 2 and the data represented in Table 5. Table 5 elucidates the findings. At all sampling locations, the surface water of the Almaty region was classified as class 3, indicative of moderately polluted water. Threshold values indicative of acceptable water quality were recorded for the elements copper, aluminum, and manganese.

The computed Water Quality Index (WQI) for all sampling points is enumerated in Table 5. As illustrated in Table 5, the WQI ranges from 9.3 (point 11) to 22.3 (point 16), which, as correlated in Table 3, signifies “Excellent water quality”. Consequently, the weighted arithmetic index effectively characterizes the surface water of Almaty City based on the concentration of chemical constituents deemed suitable for potable purposes.

### 4.3. Assessment of Carcinogenic and Non-Carcinogenic Risks

The minimum, maximum and average CDI values ((µg/(kg × day)) calculated according to Formulas (5) and (6) are presented in Table 6.

As can be seen from Table 6, the most assimilated elements through the skin, in descending order, are as follows: Fe > Ni > U > Mo > Cr > Cu > As > Co > Cd > Pb > Mn > Zn. When swallowed and taken orally (oral), the order is as follows: Fe > Mn > U > Zn > Mo > Ni > Cu > Cr > As > Co > Pb > Cd.

The results of the assessment of non-carcinogenic (HI) and carcinogenic (CR) risks (Formulas (7)–(10)) are presented in Table 7.

Table 7 shows that the HI under all conditions of toxic elements entering the human body with water meets the criterion HI < 1, meaning there is no non-carcinogenic risk.

Risks associated with the intake of carcinogenic elements in contact with the skin are at the level of 7.79 × 10^−9^–1.2 × 10^−8^, which corresponds to the criterion of no risk. CR in cases of accidental ingestion of water during bathing is generally acceptable, but at points 5 and 7, it is at the level of acceptable risk (1 × 10^−6^ < Cringestion < 1 × 10^−4^). The highest carcinogenic risk occurs when drinking water, when 8.23 × 10^−4^ < Croral < 1.52 × 10^−2^, which indicates an extremely high carcinogenic risk for an adult.

**Figure 2:**
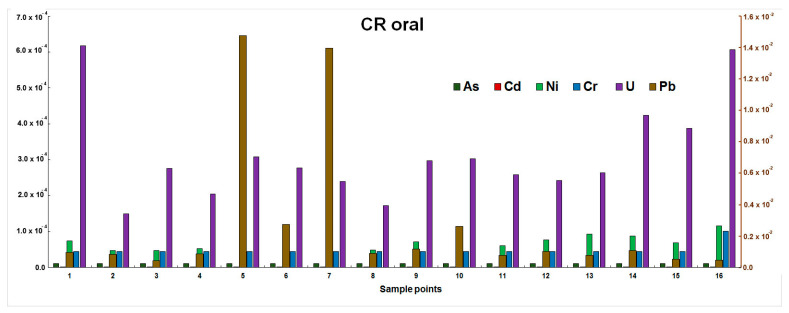
CR oral level for As, Cd, Ni, Cr, U (left scale) and Pb (right scale).

The highest contribution to carcinogenic risk is made by the presence of Pb and U in water (Figure 2).

The concentrations of contaminants in CRoral for As are quantified at 1.05 × 10^−5^; Cd: 1.05 × 10^−7^; Ni: 4.69 × 10^−5^–1.16 × 10^−4^; Cr: 4.4 × 10^−5^–1.01 × 10^−4^; U: 1.49 × 10^−4^–6.17 × 10^−4^; Pb: 4.45 × 10^−4^–1.48 × 10^−2^. The most significant risk, approximately 10^−2^, is associated with sampling sites 5 (Malaya Almatinka) and 7 (Bolshaya Almatinka), where the elevated average seasonal concentrations of lead in water, exceeding 3 μg/L, were identified (refer to Table 5). Furthermore, the peak concentrations of this element were observed in the spring season, with recorded values of 15.5 and 14.7 μg/L at sampling points 5 and 7, respectively. A notable feature of these sampling locations is their proximity to a prominent urban thoroughfare. It is plausible that the heightened lead concentrations in spring can be attributed to the accumulation of lead within the snow cover during the winter months, as cited in various academic publications [46,47]. As the snow thaws in spring, the resultant runoff facilitates the transport of contaminants into the riverine systems.

Consequently, the adherence of water quality to sanitary and hygienic standards (MPC), as delineated in Section 4.2, constitutes only one metric for evaluating its appropriateness for human consumption. Notwithstanding the characterization of water sourced from the rivers of Almaty quality as “suitable for drinking purposes” based on Water Quality Index (WQI) assessments, the contents of As, Ni, Cr, U, and Pb in it exceed the thresholds for human consumption according to carcinogenic risk evaluations.

Upon the introduction of arsenic into the human body, it exerts detrimental effects on the integumentary system, central nervous system, neurological and cardiovascular systems, gastrointestinal tract, immune response, and hormonal regulation. Lead adversely impacts the central nervous system, reproductive health, fetal development, and hormonal balance. Nickel influences enzymatic functions, arterial pressure, and skin pigmentation disorders such as vitiligo. Chromium affects the endocrine system, hematopoiesis, and the metabolic processes within the organism. Uranium, in addition to its radiotoxic characteristics, exerts effects on renal function and alters blood biochemical parameters.

## 5. Conclusions

The study utilized ICP-MS and ICP-AES to analyze surface water elemental composition in Almaty during 2023. Several sites exhibited lead, uranium, nickel, and chromium levels surpassing safety thresholds for human consumption per carcinogenic risk evaluations. While the water quality was deemed “moderately polluted”, the presence of these toxic elements indicates significant long-term health risks, notably carcinogenic concerns from ingestion and skin contact.

The detection of elevated heavy metal levels, particularly Pb and U, is vital due to their association with severe health consequences, including neurological impairment, renal issues, and cancer. Seasonal contamination fluctuations were noted, with spring runoff likely exacerbating concentrations due to melting snow and existing pollutants.

Despite the Water Quality Index (WQI) deeming the water safe for consumption, the carcinogenic risk assessment reveals considerable long-term risks from these elements, suggesting that current standards may inadequately safeguard against carcinogenic effects. This disparity emphasizes the necessity for stricter regulatory measures that consider cumulative exposure risks.

It is advisable to revise local water quality standards to incorporate both immediate toxicity and long-term carcinogenic risks for elements like lead and uranium. Enhanced and more frequent seasonal monitoring is recommended to effectively manage contaminant concentration fluctuations. Public advisories should be issued for communities dependent on these water sources, particularly during heightened contamination periods. Improvements in stormwater management and industrial waste treatment are also essential to mitigate heavy metal runoff into water bodies. This study highlights the critical need to realign current regulatory frameworks with health risk assessments to ensure better long-term protection of human health.

## Figures and Tables

**Table 1:** Characteristics of the water use classes.

Water Quality Class	Characterization of Water Use Categories
Class 1 (very good quality)	Surface waters in which there are no changes (or they are very small) in physico-chemical and biological quality values. Concentrations of pollutants do not affect the functioning of aquatic ecosystems and are not harmful to human health. Surface waters of this class are intended for all types (categories) of water use.
Class 2 (good quality)	Surface waters that are insignificantly affected by human activity and are suitable for all types (categories) of water use. Simple water treatment methods are required for domestic and drinking water use.
Class 3 (moderately contaminated)	Surface waters whose physico-chemical and biological values moderately deviate from the natural background of water quality due to human activities. Moderate signs of disturbance of ecosystem functioning are registered. Waters of this class are undesirable for salmonid fish farming, and more effective treatment methods are required for their use for domestic and drinking purposes. For all other categories of water use (recreation, irrigation, industry), the species of this class are suitable without limitation.

**Table 2:** Threshold values of the chemical element content for each quality class (µg/L).

Indicator	Quality Class of Water Bodies					
	1	2	3	4	5	6
Al	40	40	500	500	500	>500
Be	0.1	0.2	0.2	2	4	>4
Fe	100	100	300	500	500	>500
Ca	180,000	180,000	170,000	150,000	150,000	180,000
Mg	≤20,000	20,000	60,000	≤100,000	100,000	>100,000
Na	120,000	200,000	200,000	200,000	200,000	>200,000
TDS	≤1,000,000	1,000,000	1,300,000	1,500,000	≤2,000,000	>2,000,000
K	50,000	50,000	50,000	<100,000	100,000	>100,000
Mn	10	10	100	200	300	>300
Cd	5	5	25	125	125	>125
Pb	120	600	600	1000	1000	>1000
Hg	0.1	0.5	1.0	1.0	1.0	>1.0
Ni	10	25	50	100	100	>100
Cu	2	2	2000	2000	2400	>2400
Co	10	10	100	100	100	>100
Cr	100	100	55,000	55,000	55,000	>55,000
As	50	50	80	100	100	>100

**Table 3:** Water quality rating in accordance with the WQI.

WQI Value	Water Quality Rating
0–25	Excellent water quality
25–50	Good water quality
51–75	Poor water quality
76–100	Very poor water quality
More than 100	Unsuitable for drinking purposes

**Table 4:** Parameters for the risk level calculation.

Parameters	Values	References
IR (L/day)	0.05 (ingestion); 2.2 (oral)	[28]
EF (day/year)	45 (ingestion, dermal); 365 (oral)	[28]
ED (year)	30	[28]
BW (kg)	70	[26,28,29]
AT (day)	10,950 (ingestion, dermal); 25,550 (oral)	[28]
SA (sm^2^)	18,000	[26,29]
Kp (cm/h)	Co, Ni (0.004); Pb (0.0001); Cr (0.003); As, Cd, Cu, Fe, Mo, U, Mn (0.001)	[26]
ET (h/day)	1	[28]
CF (L/cm^3^)	1/1000	[26,29]
RfDoral/ingestion (µg/(kg × day)	As (0.3); Co(0.3); Cd (1); Cu (40); Mo (5.0); Ni (20); Pb (3.5); U (3); Cr (3); Fe (700); Mn (140); Zn (300)	[26,30,31,32]
RfDderm (µg/(kg × day)	As (0.285); Co (0.0003); Cd (0.025); Cu (12); Mo (1.9); Ni (5.4); Pb (0.42); Cr (0.015); Fe (300); Mn (0.8); Zn (60)	[26,30,31,32]
Sforal (µg/kg/day)^−1^	As (1500); Cd (15,000); Ni (840); Pb (8.5); Cr (500)	[26,31,32,33,34]
Sfderm (µg/kg/day)^−1^	As (3660); Cd (20,000); Cr (20,000)	[26,31]
SF_U_ (Risk/pCi)	U (6.4 × 10^−11^)	[34]

**Table 5:** Average seasonal contents and standard deviations of chemical elements in surface water samples of Almaty city.

Indicator	1	2	3	4	5	6	7	8	9	10
	Mean ± SD, µg/L									
As	<0.5	<0.5	<0.5	<0.5	<0.5	<0.5	<0.5	<0.5	<0.5	<0.5
Be	<0.03	<0.03	<0.03	<0.03	<0.03	<0.03	<0.03	<0.03	<0.03	<0.03
Co	0.100 ± 0.050	0.070 ± 0.006	0.070 ± 0.003	0.110 ± 0.090	0.130 ± 0.060	0.090 ± 0.030	0.080 ± 0.010	0.070 ± 0.010	0.100 ± 0.040	0.100 ± 0.050
Cd	<0.05	<0.05	<0.05	<0.05	<0.05	<0.05	<0.05	<0.05	<0.05	<0.05
Cu	2.4 ± 2.9	2.5 ± 3.6	2.6 ± 3.9	1.6 ± 1.9	3.2 ± 3.9	3.2 ± 4.7	1.4 ± 0.9	4.0 ± 6.6	4.3 ± 5.3	3.3 ± 5.0
Li	3.7 ± 3.1	1.2 ± 0.3	1.5 ± 0.9	1.7 ± 1.3	1.3 ± 0.3	1.2 ± 0.2	1.2 ± 0.2	1.1 ± 0.2	1.1 ± 0.2	1.3 ± 0.4
Mo	8.5 ± 1.8	5.5 ± 1.8	7.5 ± 1.0	6.0 ± 2.4	6.4 ± 2.0	7.7 ± 0.8	7.1 ± 0.7	5.8 ± 2.1	6.8 ± 1.6	6.5 ± 1.0
Ni	2.0 ± 0.8	1.3 ± 0.3	1.3 ± 0.2	1.4 ± 0.9	2.0 ± 1.0	1.6 ± 0.6	1.6 ± 0.6	1.3 ± 0.6	1.9 ± 1.0	1.8 ± 1.1
Pb	0.26 ± 0.14	0.23 ± 0.25	0.12 ± 0.07	0.24 ± 0.22	3.99 ± 7.67	0.74 ± 0.80	3.78 ± 7.06	0.24 ± 0.22	0.32 ± 0.15	0.71 ± 1.14
Se	<3	<3	<3	<3	<3	<3	<3	<3	<3	<3
U	14.6 ± 8.2	3.5 ± 1.0	6.5 ± 2.2	4.8 ± 3.1	7.3 ± 2.0	6.5 ± 1.5	5.6 ± 1.8	4.1 ± 1.9	7.0 ± 0.7	7.1 ± 1.2
Hg	<0.1	<0.1	<0.1	<0.1	<0.1	<0.1	<0.1	<0.1	<0.1	<0.1
Al	82 ± 90	51 ± 38	48 ± 35	86 ± 61	81 ± 91	52 ± 53	62 ± 35	62 ± 45	122 ± 24	87 ± 51
Ba	28.6 ± 5.5	11.5 ± 3.9	15.6 ± 8.4	31.9 ± 40.9	32.9 ± 10.7	15.3 ± 4.7	20.5 ± 11.2	24.8 ± 21.9	111.0 ± 140	41.1 ± 17.9
Cr	<0.7	<0.7	<0.7	<0.7	<0.7	<0.7	<0.7	<0.7	<0.7	<0.7
Fe	52.0 ± 48.2	39.1 ± 34.8	39.2 ± 18.4	53.9 ± 35.9	79.1 ± 56.9	49.4 ± 42.7	118 ± 125	32.7 ± 23.8	99.1 ± 44.3	43.3 ± 21.8
Mn	6.3 ± 6.5	5.5 ± 3.9	7.2 ± 6.5	12.9 ± 15.1	41.7 ± 51.2	4.8 ± 4.9	10.9 ± 7.8	17.1 ± 8.1	19.1 ± 10.0	21.3 ± 6.5
Sr	190 ± 82	86 ± 12	107 ± 17	132 ± 93	146 ± 44	128 ± 23	124 ± 23	91 ± 24	119 ± 11	154 ± 83
V	<1	<1	<1	<1	<1	<1	<1	<1	<1	<1
Zn	7.0 ± 5.2	5.3 ± 5.2	4.7 ± 2.7	6.4 ± 5.6	6.3 ± 4.0	14.1 ± 15.9	5.7 ± 3.5	4.7 ± 1.8	7.0 ± 4.8	5.1 ± 3.4
Ca	34,600 ± 13,400	19,600 ± 1900	24,200 ± 3800	26,600 ± 13,100	29,400 ± 10,600	28,300 ± 4600	27,400 ± 4900	20,900 ± 2600	24,100 ± 2100	29,000 ± 12,500
K	1900 ± 900	1350 ± 370	1100 ± 500	1700 ± 1020	1400 ± 520	1000 ± 100	1300 ± 500	1400 ± 300	1300 ± 300	1400 ± 400
Mg	5300 ± 3400	2240 ± 340	3000 ± 500	3500 ± 2600	3500 ± 1500	3500 ± 700	3600 ± 600	2700 ± 1100	3100 ± 800	4600 ± 3700
Na	10,200 ± 9500	6000 ± 7800	3900 ± 1000	38,100 ± 73,000	5400 ± 4200	6100 ± 3600	5200 ± 1200	3600 ± 2900	4600 ± 3000	7600 ± 8800
SO_4_^2−^	<20,000	<20,000	<20,000	<20,000	<20,000	<20,000	<20,000	<20,000	<20,000	<20,000
Cl^−^	5750 ± 1500	6250 ± 2500	6000 ± 2000	58,750 ± 107,500	7750 ± 5500	7000 ± 2309	5750 ± 1500	<5000	6000 ± 2000	10,000 ± 10,000
TDS	144,800 ± 60,200	88,500 ± 15,000	99,000 ± 13,600	199,500 ± 251,105	117,500 ± 45,420	88,500 ± 46,500	103,700 ± 10,300	87,800 ± 19,400	88,000 ± 26,000	126,300 ± 81,000
Water quality class with polluted elements	3 Cu. Al	3 Cu. Al	3 Cu. Al	3 Al. Mn	3 Cu. Al. Mn	3 Cu. Al	3 Al. Mn	3 Cu. Al. Mn	3 Cu. Al. Mn	3 Cu. Al. Mn
WQI	16.8	5.36	8.15	8.81	15.3	8.32	11.1	7.33	16.2	11.7
**Indicator**	**11**	**12**	**13**	**14**	**15**	**16**	**MPC (Drink Water)**	**Hazard Class**	**LOD**	**LOQ**
	**Mean ± SD, µg/L**									
As	<0.5	<0.5	<0.5	<0.5	<0.5	<0.5	50	2	0.5	1.3
Be	<0.03	<0.03	<0.03	<0.03	<0.03	<0.03	0.2	1	0.03	0.08
Co	0.080 ± 0.020	0.110 ± 0.060	0.130 ± 0.090	0.150 ± 0.140	0.120 ± 0.060	0.240 ± 0.070	100	2	0.07	0.2
Cd	<0.05	<0.05	<0.05	<0.05	<0.05	<0.05	1	2	0.05	0.1
Cu	3.4 ± 5.3	4.0 ± 4.4	2.7 ± 3.6	2.7 ± 2.1	2.1 ± 2.0	2.5 ± 1.3	1000	3	0.5	1.3
Li	1.3 ± 0.3	1.6 ± 0.8	3.1 ± 1.5	4.3 ± 5.1	2.8 ± 2.4	8.8 ± 4.7	30	2	0.2	0.5
Mo	7.3 ± 0.7	9.0 ± 4.1	11.7 ± 8.3	7.6 ± 2.3	8.9 ± 3.7	10.7 ± 4.8	250	2	0.3	0.8
Ni	1.6 ± 0.6	2.0 ± 0.8	2.5 ± 1.0	2.3 ± 1.1	1.8 ± 0.6	3.1 ± 1.3	100	3	0.5	1.3
Pb	0.21 ± 0.19	0.28 ± 0.19	0.21 ± 0.16	0.29 ± 0.23	0.15 ± 0.09	0.13 ± 0.07	30	2	0.05	0.13
Se	<3	<3	<3	<3	<3	<3	10	2	3	7.5
U	6.1 ± 1.2	5.7 ± 1.1	6.2 ± 3.5	10.0 ± 5.3	9.1 ± 3.0	14.3 ± 4.7	30	1	0.03	0.1
Hg	<0.1	<0.1	<0.1	<0.1	<0.1	<0.1	0.5	1	0.1	0.3
Al	64 ± 35	70 ± 67	97 ± 53	121 ± 60	67 ± 38	30 ± 23	500	2	3	8
Ba	28.3 ± 22.6	37.4 ± 21.9	42.8 ± 41.0	57.9 ± 28.8	40.9 ± 36.1	52.1 ± 8.5	100	2	0.5	1.3
Cr	<0.7	<0.7	<0.7	<0.7	2.0 ± 0.6	<0.7	50	3	0.7	1.8
Fe	44.4 ± 11.3	45.7 ± 37.2	54.9 ± 22.0	80.0 ± 34.0	48.9 ± 15.3	21.7 ± 5.9	300	3	0.4	1.0
Mn	16.5 ± 12.0	26.5 ± 9.9	23.6 ± 19.5	27.7 ± 13.6	16.0 ± 11.3	16.1 ± 8.9	100	3	0.5	1.3
Sr	138 ± 43	184 ± 117	422 ± 301	239 ± 124	350 ± 369	583 ± 330	7000	2	0.5	1.3
V	<1	<1	<1	<1	<1	<1	100	3	1	2.5
Zn	5.4 ± 3.9	13.5 ± 14.4	4.5 ± 2.6	8.2 ± 4.8	4.0 ± 2.6	12.8 ± 15.7	5000	3	0.7	1.8
Ca	28,700 ± 5800	34,000 ± 12,000	48,550 ± 22,100	36,300 ± 18,700	40,600 ± 16,400	54,400 ± 21,700	30,000		10	30
K	1200 ± 470	1500 ± 700	1800 ± 600	1650 ± 790	2030 ± 1600	2620 ± 1110	n.r. *		15	40
Mg	4400 ± 1500	5200 ± 3500	7600 ± 4600	6250 ± 5600	13,200 ± 16,700	21,600 ± 12,800	20,000		30	80
Na	8000 ± 6800	7000 ± 4200	16,000 ± 15,000	13,000 ± 13,000	23,000 ± 32,000	32,800 ± 20,500	200,000	2	10	30
SO_4_^2−^	<20,000	<20,000	<20,000	<20,000	<20,000	103,000 ± 26,000	500,000	4	20,000	50,000
Cl^−^	12,500 ± 12,500	15,500 ± 18,400	43,300 ± 34,100	19,250 ± 14,900	25,000 ± 30,900	30,000 ± 13,000	350,000	4	5000	13,000
TDS	128,500 ± 42,000	144,000 ± 81,400	260,800 ± 139,600	193,500 ± 92,200	255,800 ± 238,000	366,800 ± 181,500	1,000,000			
Water quality class with polluted elements	3 Cu. Al. Mn	3 Cu. Al. Mn	3 Cu. Al. Mn	3 Cu. Al. Mn	3 Cu. Al. Mn	3 Cu. Mn				
WQI	9.30	10.7	12.5	17.6	13.5	22.3				

*—not regulated.

**Table 6:** Minimum, maximum and average CDI values ((µg/(kg × day)).

Chemical Element	CDIdermal			CDIingestion			CDIoral		
	Min	Max	Mean	Min	Max	Mean	Min	Max	Mean
As	1.59×10^−5^	1.59 × 10^−5^	1.59 × 10^−5^	4.40 × 10^−5^	4.40 × 10^−5^	4.40 × 10^−5^	1.57 × 10^−2^	1.57 × 10^−2^	1.57 × 10^−2^
Co	8.34 × 10^−6^	3.43 × 10^−5^	1.39 × 10^−5^	5.79 × 10^−6^	2.38 × 10^−5^	9.67 × 10^−6^	2.07 × 10^−3^	8.51 × 10^−3^	3.45 × 10^−3^
Cd	1.59 × 10^−6^	1.59 × 10^−6^	1.59 × 10^−6^	4.40 × 10^−6^	4.40 × 10^−6^	4.40 × 10^−6^	1.57 × 10^−3^	1.57 × 10^−3^	1.57 × 10^−3^
Cu	1.59 × 10^−5^	6.89 × 10^−5^	4.06 × 10^−5^	4.40 × 10^−5^	1.91 × 10^−4^	1.13 × 10^−4^	1.57 × 10^−2^	6.83 × 10^−2^	4.03 × 10^−2^
Mo	1.50 × 10^−4^	2.69 × 10^−4^	2.19 × 10^−4^	4.17 × 10^−4^	7.48 × 10^−4^	6.07 × 10^−4^	1.49 × 10^−1^	2.67 × 10^−1^	2.17 × 10^−1^
Ni	1.60 × 10^−4^	4.74 × 10^−4^	2.72 × 10^−4^	1.11 × 10^−4^	3.29 × 10^−4^	1.89 × 10^−4^	3.97 × 10^−2^	1.18 × 10^−1^	6.73 × 10^−2^
Pb	1.59 × 10^−7^	1.17 × 10^−6^	2.82 × 10^−7^	4.40 × 10^−6^	3.24 × 10^−5^	7.82 × 10^−6^	1.57 × 10^−3^	1.16 × 10^−2^	2.79 × 10^−3^
U	1.17 × 10^−4^	5.78 × 10^−4^	2.53 × 10^−4^	3.24 × 10^−4^	1.60 × 10^−3^	7.02 × 10^−4^	1.16 × 10^−1^	5.73 × 10^−1^	2.51 × 10^−1^
Cr	6.66 × 10^−5^	1.52 × 10^−4^	7.19 × 10^−5^	6.16 × 10^−5^	1.41 × 10^−4^	6.66 × 10^−5^	2.20 × 10^−2^	5.03 × 10^−2^	2.38 × 10^−2^
Fe	2.98 × 10^−4^	9.51 × 10^−3^	1.55 × 10^−3^	8.27 × 10^−4^	2.64 × 10^−2^	4.30 × 10^−3^	2.95 × 10^−1^	9.43	1.54
Mn	3.46 × 10^−9^	4.79 × 10^−8^	2.22 × 10^−8^	1.73 × 10^−4^	2.40 × 10^−3^	1.11 × 10^−3^	6.17 × 10^−2^	8.55 × 10^−1^	3.96 × 10^−1^
Zn	2.73 × 10^−9^	3.91 × 10^−8^	8.30 × 10^−9^	2.28 × 10^−4^	3.26 × 10^−3^	6.92 × 10^−4^	8.12 × 10^−2^	1.16	2.47 × 10^−1^

**Table 7:** Result of the calculation of carcinogenic and non-carcinogenic risks for different sampling points.

Sampling Point	HIdermal	HIingestion	HIoral	HI	CRdermal	CRingestion	CRoral	CR
1	0.05	8.11 × 10^−4^	0.29	0.34	7.74 × 10^−9^	3.07 × 10^−6^	1.70 × 10^−3^	1.70 × 10^−3^
2	0.04	4.21 × 10^−4^	0.15	0.19	7.74 × 10^−9^	2.64 × 10^−6^	1.09 × 10^−3^	1.09 × 10^−3^
3	0.04	5.42 × 10^−4^	0.19	0.23	7.74 × 10^−9^	1.55 × 10^−6^	8.23 × 10^−4^	8.25 × 10^−4^
4	0.05	4.85 × 10^−4^	0.17	0.23	7.74 × 10^−9^	2.75 × 10^−6^	1.18 × 10^−3^	1.18 × 10^−3^
5	0.06	6.89 × 10^−4^	0.25	0.31	7.74 × 10^−9^	4.17 × 10^−5^	1.52 × 10^−2^	1.52 × 10^−2^
6	0.04	5.71 × 10^−4^	0.20	0.25	7.74 × 10^−9^	8.01 × 10^−6^	3.13 × 10^−3^	3.14 × 10^−3^
7	0.04	6.12 × 10^−4^	0.22	0.26	7.74 × 10^−9^	3.95 × 10^−5^	1.43 × 10^−2^	1.44 × 10^−2^
8	0.04	4.51 × 10^−4^	0.16	0.20	7.74 × 10^−9^	2.82 × 10^−6^	1.17 × 10^−3^	1.18 × 10^−3^
9	0.05	5.79 × 10^−4^	0.21	0.26	7.74 × 10^−9^	3.64 × 10^−6^	1.59 × 10^−3^	1.59 × 10^−3^
10	0.05	5.80 × 10^−4^	0.21	0.26	7.74 × 10^−9^	7.71 × 10^−6^	3.05 × 10^−3^	3.06 × 10^−3^
11	0.04	5.40 × 10^−4^	0.19	0.23	7.74 × 10^−9^	2.47 × 10^−6^	1.13 × 10^−3^	1.14 × 10^−3^
12	0.05	5.82 × 10^−4^	0.21	0.26	7.74 × 10^−9^	3.26 × 10^−6^	1.40 × 10^−3^	1.40 × 10^−3^
13	0.06	6.44 × 10^−4^	0.23	0.29	7.74 × 10^−9^	2.59 × 10^−6^	1.18 × 10^−3^	1.18 × 10^−3^
14	0.07	6.97 × 10^−4^	0.25	0.32	7.74 × 10^−9^	3.46 × 10^−6^	1.65 × 10^−3^	1.65 × 10^−3^
15	0.06	6.65 × 10^−4^	0.24	0.29	7.74 × 10^−9^	1.88 × 10^−6^	1.05 × 10^−3^	1.05 × 10^−3^
16	0.11	9.16 × 10^−4^	0.33	0.44	1.20 × 10^−8^	1.98 × 10^−6^	1.30 × 10^−3^	1.30 × 10^−3^

## Data Availability

Data Availability Statement: Our dataset has been assigned two important links: a reviewer URL: https://datadryad.org/stash/share/5Izd9F9bPne9Z1PcQ1K6MhWkgZ_zs7DJkQFTKrSNSt4 (accessed on 2 September 2024); a unique digital object identifier (DOI): https://doi.org/10.5061/dryad.mpg4f4r80 (accessed on 2 September 2024).
